# Sentence Context and Word-Picture Cued-Recall Paired-Associate Learning Procedure Boosts Recall in Normal and Mild Alzheimer's Disease Patients

**DOI:** 10.1155/2018/7401465

**Published:** 2018-04-17

**Authors:** Rosario Iodice, Juan José García Meilán, Juan Carro Ramos, Jeff A. Small

**Affiliations:** ^1^Catholic University of Pereira, Risaralda, Colombia; ^2^Institute of Neurosciences of Castilla y León, University of Salamanca, Salamanca, Spain; ^3^Department of Psychology, University of Salamanca, Salamanca, Spain; ^4^School of Audiology and Speech Sciences, The University of British Columbia, Vancouver, BC, Canada

## Abstract

**Introduction:**

The aim of this study was to employ the word-picture paradigm to examine the effectiveness of combined pictorial illustrations and sentences as strong contextual cues. The experiment details the performance of word recall in healthy older adults (HOA) and mild Alzheimer's disease (AD). The researchers enhanced the words' recall with word-picture condition and when the pair was associated with a sentence contextualizing the two items.

**Method:**

The sample was composed of 18 HOA and 18 people with mild AD. Participants memorized 15 pairs of words under word-word and word-picture conditions, with and without a sentence context. In the paired-associate test, the first item of the pair was read aloud by participants and used to elicit retrieval of the associated item.

**Results:**

The findings suggest that both HOA and mild-AD pictures improved item recall compared to word condition such as sentences which further enabled item recall. Additionally, the HOA group performs better than the mild-AD group in all conditions.

**Conclusions:**

Word-picture and sentence context strengthen the encoding in the explicit memory task, both in HOA and mild AD. These results open a potential window to improve the memory for verbalized instructions and restore sequential abilities in everyday life, such as brushing one's teeth, fastening one's pants, or drying one's hands.

## 1. Introduction

Alzheimer's disease (AD) is a progressive neurodegenerative disorder that includes deterioration across many cognitive domains. The most commonly reported problem in the early stages of AD is progressive memory loss, in particular episodic recent memory impairment that produces serious difficulty to encode and consolidate new information, resulting from medial temporal lobe (MTL) neural degeneration [1].

Despite the loss of episodic memory in the context of MTL degeneration, there is a relative preservation of semantic knowledge in the AD patients [2, 3] which could represent an opportunity to improve episodic memory, because there exists a relation between semantic and episodic memory. A proof of this is paired associated learning (PAL) in which the encoding of a new episodic memory is affected by the degree of association between the two words (semantic memory) [4]. Generally, the verbal PAL task has been used as an instrument for assessing explicit episodic memory performance in healthy older people, with mild cognitive impairment (MCI) and with early AD [5–8]. In this study, we used PAL task in a new way, not as an assessment instrument but like strategic memory control processes to investigate together the strategic component of episodic memory (organization and manipulation of information during encoding, storage, or retrieval) [9] and the types of mediators (pairs of words, the word followed by an image, and sentence-generation strategies) that determine individuals' encoding performance. The mediator is an aid or strategy that is used to link together the items to be remembered [10]. In these terms, the encoding operation constitutes a control process that can promote the increased levels of recall performance [11].

One possible mediator that has shown improvements in memory performance in healthy young [12] and older adults [13] and has promoted the modality congruency effect (this refers to the congruency between the modality of presentation and the modality of recall) is the interactive imagery strategy. Decades of research (over 50 years) have found that pictures are retained in memory better than words are (picture superiority effect). There exists a wide range of cognitive psychology studies that have shown that pictures enhance recollection compared with words in healthy young and older adults [14]. Consistent empirical finding shows that when a word can evoke an image or have a semantic relation with a pictorial representation, verbal and image codes are stored in interconnected symbolic system memory [15, 16]. The effectiveness of a pictorial representation can improve memory for words, because semantic elaboration enhances the pictorial superiority effect [17, 18]. One theory that explains the picture superiority effect proposes that the pictures allow a deeper and more elaborate conceptual processing than solely words do (semantic processing account) [19, 20]. Several authors have found that individuals with amnesic mild cognitive impairment (aMCI) and mild AD can enhance memory by extracting the conceptual meaning from pictures [21] because they enhance conceptual fluency [22, 23]. Furthermore, the picture enhances perceptual fluency and improves the sense of familiarity [24], which in turn improves accuracy and reduces false recognition [25] compared with words.

Despite that, the widespread limitation found in the experimental designs in which the purpose has been to demonstrate the effectiveness of pictorial representation to improve the memory for words in aMCI or mild AD is that the majority of experimental tasks are based on recognition memory [26].

In this work, we want to propose a sentence's linguistic structure combined with pictorial representation, as a possible mediator to improve the process of encoding new items in mild-AD patients and promote explicit memory retrieval for words. We consider that the linguistic structure of a sentence can be thought as an autopoietic system that receives feedback from itself (in terms of linguistic structure) strengthening the unity between the elements (words). The phrase is inserted in a network that allows it to be activated in different ways (lexical, semantic, and visual). The diversification of activation supports the duration of the memory and therefore access to it [27–29]. There is little research that has directly examined the possibility to use sentence as a mediator in mild AD; generally, the sentence has been used to assess the problem in verbal comprehension in mild AD, as we extensively found in literature [30–33]. We consider that it not is the only form to use the linguistic property of the sentence, because it can be converted into an adequate strategy (mediator) that could improve the process of encoding new items in mild-AD patients, because the sentence favors a sufficient semantic richness which allows it to improve the retrieval of the information preview learned [34]. Sentence processing is a natural task, and it can take advantage of sentence cues when processing the meaning, especially when the sentence context primes certain aspects of a target word's meaning. Retrieval of words in the sentences is facilitated when there is a unique cue-target association that reduces the interference and reliable access to the information. Moreover, a sentential context provides a semantic structure for integration of the meaning across the words and allows the formation of a comprehensive mental image [35, 36].

According to these purposes, it is important to take into account that sentence contexts activate both semantic and lexical hierarchical networks in which stimulation of a concept's representation activates other concept nodes associated with the original concept [37, 38], priming the target words included in a phrase [39] and improving the encoding and retrieval of information [40]. Several authors attributed the advantages of sentence context to an activation of automatic processes, especially when a word is thought to prime other semantically related concepts and boost the processing of a target word [41, 42]. Furthermore, the semantic content of a sentence can provide a semantic structure for the integration of meaning across the words and thus allows the formation of a comprehensive mental image [41].

To further enhance encoding, some authors suggest combining verbal and pictorial stimuli to activate a presemantic level of information that interacts with the episodic and semantic systems. Paivio et al. [43] have shown that the ability to generate interactive visual images facilitates verbal PAL performance, independently from the effect of stimulus relatedness. That is, the interconnectivity of words and images facilitates access to a common conceptual memory storage area [18, 44]. Cherry et al. [45] carried out a study with younger and older adults, where the encoding stimuli consisted of sets of 16 sentences with pictorial illustrations (e.g., The dusty man held the rope), and the retrieval stimuli consisted of “what” question sentences (e.g., “What did the dusty man do?”). They found that the combination of verbal and visual supports allowed retrieval of the main point of the sentences and improved recall performance.

Although these strategies are based on known cognitive paradigms and have a long path of experimentation when they are combined with pictorial and verbal material, the greatest limitation found in previous research is that mild AD is able to extract only the gist from pictorial information, but they can remember the information in verbatim form. The mnemonic strategy is not easy to learn because they are often complex and have a high level of artificiality that generates difficulties using them in daily life. Furthermore, when the therapists interact with mild-AD patients, they found serious difficulties to promote the memorization of complex information such as sentences; generally, the mnemonic method is limited to a simple and short piece of information (face nouns, date, and single words) (see [46] for limitations of mnemonics in clinical applications). In this work, we propose the use of the combinations of two known strategies according to which the sentence context enriched by pictorial illustration represents a form of environmental support where the activation of a sentential semantic network more robustly connects to a visual representation [14, 15, 39, 47]. We consider it important to investigate the relationship between semantic/conceptual (sentences) and visual perception conditions (pictures) during both encoding and retrieval in mild-AD patients, because understanding this relationship would generate new reflections and suggest new forms to use and combine mnemonic strategies that favor the codification of new information.

Taking into account that it is possible to use sentences as contextual cue combined with pictorial representation, we consider that it is also possible in mild AD, because the older adults have a greater experience with sentence processing during their lives because they reprocess the verbal information every day, and they depend more heavily on a set of rich sentential context cues for recalling the information [47]. However, this may have several implications in a clinical context: on the one hand, the linguistic structure of sentences makes mnemonic techniques more natural and familiar, allowing easy use of them in everyday life and promoting the retrieval in explicit memory task.

Despite advances in our understanding of the effects of sentence context as contextual cue, there is little research and timid attempts that have directly examined the importance of the relationship between semantic/conceptual and visual perception conditions during both encoding and retrieval of items embedded in short sentences for healthy older adults (HOA) and persons with mild AD. Understanding these mechanisms would open up new directions for cognitive interventions in older adults with cognitive impairment. Therefore, the specific aim of this work, based on the theoretical models and empirical research described above, was to assess the effectiveness of encoding sentence context as a strong contextual cue (cue-target association) and different types of stimuli in HOA and persons with mild AD. In this study, we used the word-word (WW) and word-picture (WP) conditions as target items [15], comparing the encoding and retrieval of the target item (second item of the pair) in the presence or absence of a sentence contextual cue. Our first hypothesis was that the WP condition would yield a higher recall rate of second pair items compared to the WW condition. Our second hypothesis was that sentence context would improve the recall of the second pair items, in both WW and WP conditions relative to the null sentence context condition. Our third prediction was that the first and second hypotheses would be confirmed for both HOA and mild-AD participants.

## 2. Method

### 2.1. Participants

Thirty-six elderly Spanish people were included in the study, forming two groups. The first group was made up of 18 patients at the State Reference Center for people with Alzheimer's disease of Spain (Salamanca). As inclusion criteria, participants had been previously diagnosed with AD (not mixed dementia) for the Spanish National Health System following the criteria of NINCDS-ADRDA [48] and whose diagnosis as AD was later confirmed according to GDS stages 2 and 3 by the center's medical and neurological service (very mild–mild cognitive decline) [49]. The mean age was 83.9 years (SD = 13.573; range 65–90), with a Mini Mental State Examination (MMSE) mean of 20.3, (SD = 2.20; range 18–24) [50]. The families of these patients provided informed consent for their participation in the study. In accordance with the Ethics Committee at the center, a form was sent out to the patients' immediate family or guardian for them to sign and return. The second group was made up of 18 people living in nursing homes who did not have a neurodegenerative pathology and who were classified as cognitively healthy older adults by a physician at the home. The mean age of the group was 80.1 (SD = 7.415; range 63–91) with a MMSE mean of 27.3 (SD = 2.024; range 24–30) [50]. The healthy older adults provided informed consent to participate in the study. We found significant differences between the two experimental groups in terms of their scores on the MMSE, *t* (32) = 6.2, *p* < .001 [5]. We confirmed that there were no significant differences between the two groups in terms of educational level, years of schooling, age, or gender distribution (see Table 1). The study excluded those participants with any depressive symptoms, measured by the Beck Depression Inventory (value > 10) [51], as well as those participants with medical backgrounds involving problems in their communication system (auditory or visual) or in their ability to read.

### 2.2. Materials

**Figure 1:**
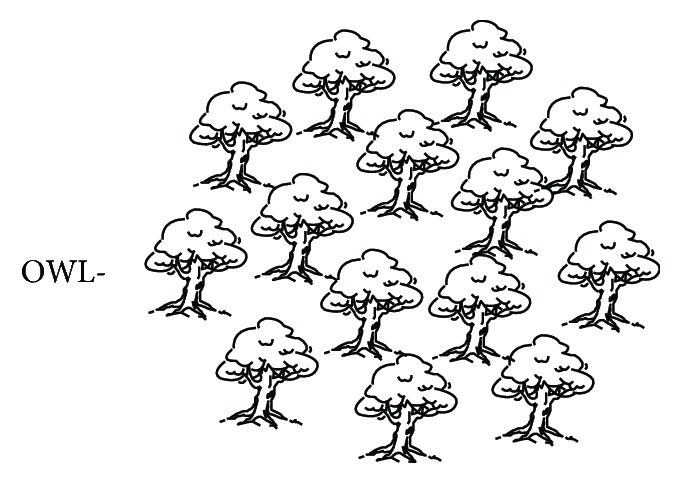
Word-picture pair (*owl-woods*) embedded in sentence context *“The owl lives in the woods.”*

We employed a database of 60 pairs of Spanish words from which we randomly created 4 lists of 15 pairs (e.g., owl-woods, umbrella-winter). We selected and matched all pairs of stimuli based on the factors' “frequency of use” and “proportion” from the normative study by Fernandez et al. [52]. Each list of stimuli contained one of the following four combinations: WW and WP each embedded in a sentence context and WW and WP each not embedded in a sentence context. The pictures for the WP condition were obtained by associating the words with corresponding Snodgrass drawings [53]. The presentation order of these stimuli was counterbalanced across the Paired Associate Learning (PAL) form and, in the embedded context condition, was embedded in a short sentence (sentence context cue), favoring the encoding and retrieval of the second (right) item of the pair. All stimuli were presented using the experimental program E-Prime® V1.1, presented on a 15^″^ screen with a resolution of 1024 × 768, and placed at a distance of 48^″^ from participants. The words were displayed in black against a white background, and the images were 3 cm in size (width 45% × height 45% in E-Prime) in the form of line drawings (see Figure 1).

### 2.3. Procedure

Each participant was tested individually and completed four 30-minute sessions, each one corresponding to one of the four list types. The order of conditions was counterbalanced across participants, and sessions were held one week apart. The individual sessions were divided into two phases, a study phase and a retrieval phase. In the study phase, 15 pairs of stimuli were presented one at a time on the computer screen for ten seconds. In the null sentence context condition, the stimuli were presented in a PAL task as two words (WW) or as a word followed by picture (WP), and the participants only read the words and named the pictures aloud. In the embedded sentence context condition, the stimuli were again presented in a PAL task (WW or WP), but, in addition, the experimenter read a corresponding sentence (e.g., after presenting the “owl-woods” pair, the experimenter read the embedded sentence: “The *owl* lives in the *woods*”; see Figure 1). In between trials, a fixation point was displayed in the center of the screen for 1 second. After the study phase (approximately 150 seconds), the participants were informed, through instructions presented on the computer screen, that the learning task had been completed and that the retrieval task was to begin immediately. During the retrieval task, the first item in the pair was presented on the screen, and the participants were asked to recall the associated second items. The participant was instructed to say “I do not remember” when he/she thought he/she was not able to recover the information. In these circumstances, the omission of the information was considered as absence of an error instead of a fault; the experimenter provided a phonological cue according to the method of “errorless learning” [54], to corroborate the presence or absence of the encoding process. For example, with the pair “umbrella (word)-winter (image),” to facilitate the recovery of the second element of the pair according to the technique of errorless learning, a phonological cue constituted by the first significant syllables of the word was provided orally, in this case “win-” (for the word winter). With this cue, the participant was invited to complete the word. If the experimenter realized that there was no clarity from the participant about the type of sounds pronounced, the letters were written on a sheet, showed to the person, and pronounced aloud again. The stimulus presentation in the recall phase was not subject to a time limit [15]. The time elapsed in this phase was managed through the recovery process described previously (inviting the participant to remember the associated pair in order to provide a phonetic cue in the absence of the memory and to finalize the recovery phase if the participant had given a correct or incorrect response). The retrieval process took no more than 5 minutes. One point was assigned for each correct retrieval, 0.5 for each retrieval by means of a phonetic cue using “errorless learning” (e.g., “wo” for “woods”), and 0 for each incorrect retrieval or persistent omission of the information. The persistent omission was considered as an omission after it provided four times the phonetic cue [55].

#### 2.3.1. Statistical Analysis

A 2 × 2 × 2 repeated-measures mixed-design analysis of variance model was performed with two within-subject variables: encoding type (WW and WP) and the presence or absence of sentence context (Presence and Absence), and one between-subject variable: participant group (mild-AD and healthy older adults). The dependent variable was the recall accuracy of the second item of each learning pair. The significance level was set at *p* < 0.05. Descriptive data for recall is shown in Table 2.

## 3. Results

**Figure 2:**
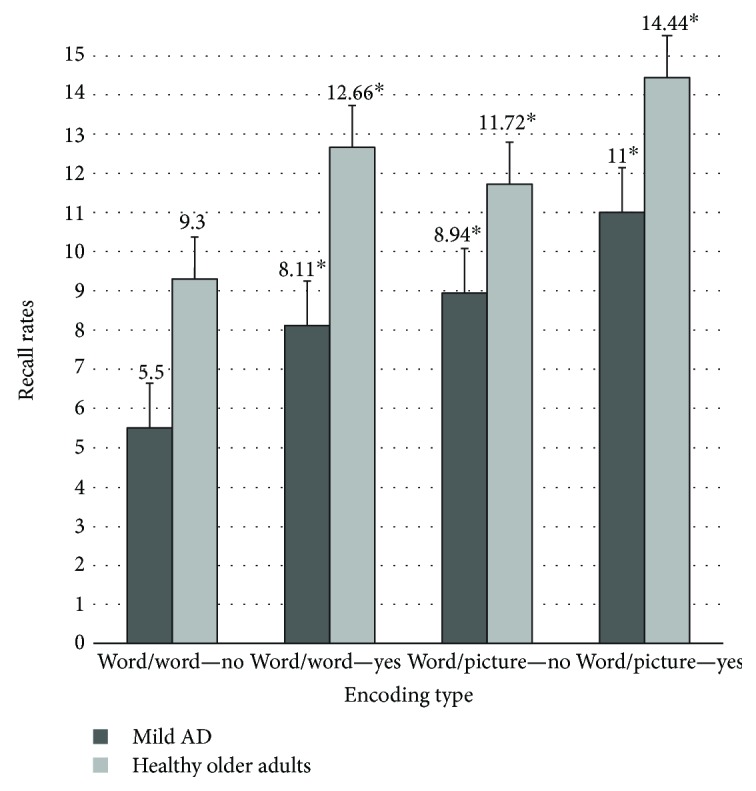
Recall rates of the second element of the pair with (yes) and without (no) sentence context, in healthy older adults and mild AD. ANOVA 2 × 2 × 2 was performed. ^∗^The significance level was set at *p* < .05. A significant main effect in word-picture compared with word-word, and in sentences compared to the null sentence context condition, in both groups was found.

A significant effect of the group factor was also observed, *F* (1, 34) = 28.645, *p* < .001, eta = .46, indicating that, as expected, the HOA had 24% greater recall success overall (mean = 12,035) than the mild-AD group had (mean = 8389, *p* < .001). Of the two manipulated variables, we found that there was a significant main effect in the type of encoding, *F* (1, 34) = 158.43, *p* < .001, eta = .82). We confirmed the superiority rate recall of WP (mean = 11,528) compared to WW as a contextual cue (mean = 8896, *p* < .001). In this case, we found a significant interaction effect between the type of encoding and group, *F* (1, 34) = 6.539, *p* < .05, eta = .16. Pairwise comparisons of the interaction confirmed that there were significant differences between the HOA (mean = 10.87) and mild-AD groups (mean = 6.81; *p* < 0.01) in the WW condition (*p* < .001, eta = .51) and in the WP condition (mean = 13.10 for HOA and mean = 9.97 for mild AD; *p* < .001, eta = .35) (see Figure 2). We also found differences between WW and WP conditions in both HOA (mean differences = 2097; *p* < 0.01, eta = .60) and mild AD (mean differences = 3167; *p* < 0.01, eta = .77). There was also a significant main effect of sentence context (*F* (1, 34) = 66.768, *p* < .001, eta = .66), in which recall was better when the items were embedded in sentences (mean = 11,556) compared to the null sentence context condition (mean = 8868, *p* < .001). There were no interactions between the use of sentences and the groups, the use of WP and sentences, and the use of WP, sentences, and groups.

## 4. Discussion

The purpose of this study was to investigate the mechanisms to improve encoding of words in the explicit memory task under WW versus WP, and sentence context versus null sentence conditions, in both HOA and persons with mild AD. The WP and sentence context improved retrieval performance in both groups. The word associated with an image (WP) improves the recall rates (17.54%) with respect to WW, with more effectiveness in people with mild AD than in HOA. Similarly, the sentence context condition improves the recall rates (17.91%) compared to the null sentence condition. We found a significant interaction effect between the type of encoding and group. This result can be in agreement with previous studies which show that the magnitude of the picture superiority effect is greater in patients than in HOA [26]. The improvement in recall rates is similar in both coding conditions. Nevertheless, the results do not show an interaction between the variables. Both types of intervention seem to act independently and therefore can be used as a way to improve memory in the elderly. There are several possible reasons for why such an interaction did not emerge. One possibility is that there was such a strong benefit of each cue by itself that any further gains by combining cues were minimal. Another reason could be related to the extra task demands associated with processing stimuli from multiple modalities (word and image) in conjunction with sentential semantic processing. Previous research has shown that processing stimuli under more complex cue conditions can actually create extra attentional demands, leading to declines in performance [56]. Despite the lack of interaction between WP and sentence context, the strong independent effects of each suggest possible intervention approaches capitalizing on the strengths of each cue type, as described below. To understand this possible application in clinical context, we take into account that there is extensive literature which highlights the difficulty of implementing mnemonic strategies for older people, especially for people with mild AD [57, 58]. One of the main criticisms about mnemonic strategies is that they are difficult to learn because they are often complex and have a high level of artificiality that generates difficulties using them in daily life. Our results indicate that, despite the memory deficit based on MTL neural degeneration that causes deficit both in episodic and in semantic memory, the HOA and mild-AD persons are able to extract the gist from pictorial information and learn with less interference because they focus on the conceptual aspect [59, 60]. The HOA individuals process the information with more amplitude as well as depth. The mild-AD persons showed that they were able to benefit from the way material was encoded at acquisition, generating a cued assistance, and that it is related to assistance provided at retrieval, enhancing recall. The results obtained in this study, although they refer to a specific section of a much wider problem (memory problem in the AD), are consistent with cross-sectional studies, where it is reported that the episodic memory impairment of mild-AD patients is characterized by encoding deficit and low retrieval performance compared to HOA [40]. At the same time, the results also show that despite the fact that mild-AD persons were unable to encode and process all the semantic properties, all participants in the present study were able to extract the gist from pictorial information and they were able to learn with less interference because they focus on the conceptual aspect of information. The results of the present study extend past research, in which it was demonstrated that mild-AD persons receive cue benefits when they are provided with instructions to encode the “to be remembered” (TBR). While in this work the patients were able to utilize cues without instructions to encode, they simply received a particular information structure (image and sentence) that generates deep encoding and provides support at retrieval [15, 61].

The results also show that sentences not only serve to assess the problem in verbal comprehension in AD patients [30–33, 62]. The sentences can be used as contextual cue, because the syntactic properties of the sentence (subject, verb, complement (SVC)) create an organized structure where the words are stringed into chunks that improve their recall performance. Several studies have shown that mild-AD patients remained structurally rich with similar syntactic structure as the HOA controls, and mild-AD patients are not impaired to determine aspects of sentence meaning, despite working memory deficits. This means that they can perform relatively normally on some semantic tasks when they are not required to search for, or intentionally manipulate, semantic information [63, 64]. The sentences can be thought as a linguistic structure that strengthens the unity between the elements (words). The simultaneous engagement of the images and words produces an overlap of codes allowing the access to a common conceptual memory storage area [41]. According to some authors, this is because the information has been drawn to a presemantic level and has interacted with episodic and semantic systems [65]. The results from the present study therefore suggest that mnemonic training, based on the WP and sentence context processing, may enhance encoding and recall by persons with mild AD. Moreover, because the WP paradigm targets lexical level processing, it would perhaps be most useful in addressing semantic memory “access” problems in mild-AD persons, whereas the deeper encoding primed by a sentence context may help persons with mild AD encode and recall a higher level of propositional information. Future research is needed to address these possibilities and to explore how mnemonics based on WP and sentence context can be effectively implemented in real-world contexts.

This study indicates that older individuals, with and without dementia, are sensitive to the semantic constraints provided by a sentence context or picture. Thus, it appears that syntactic properties of the sentence can facilitate the abilities of HOA and those with mild AD to carry out operations such as those involved in retrieval of target information [66]. There is data which indicates that patients with mild AD retain their knowledge of semantic features, but they have difficulty voluntarily accessing it because the information is disorganized [67]. The results of the present study suggest that, first of all, these individuals' performance is improved in encoding because the sentence context and picture generate sets of feature restrictions that serve to organize and integrate the words and allow the formation of a comprehensive mental image [15]. Secondly, the sentence and visual representation of the target words creates a context in which there is an accumulation of presemantic activations. This condition enables the person to better process higher-level representations of the meaning of a sentence and a picture, including relevant attributes that are necessary for promoting a deeper encoding of information [68]. Thirdly, reading a sentence requires deeper processing of its constituent words than does reading a pair of nouns [69]. Thus, our two interventions establish a meaningful relationship among its constituent words, thereby reducing the arbitrariness of a pairwise association. The sentence context can be thought of as a mnemonic strategy, whereby the whole promotes greater depth of encoding than the sum of the parts [14].

As a consequence of the discussion and conclusion of this study, we propose a speculative question: if it is possible to increase memory for words when they are embedded in sentence context, can the same pair of target words activate a semantic network that allows to retrieve a sentence in verbatim form? It helps patients to improve memory for sentences in verbatim form, providing a potential window for nonpharmacological therapies. The sentences can be thought as an autopoietic structure. In other words, as it is possible to improve the memorization of words through a sentence by using syntactic and semantic structures which activate semantic networks, in the same way, a pair of words which would have a certain semantic relationship could activate the same networks in order to favor the integral recovery of the sentence. The phrase is inserted in a network that allows it to be activated in different ways (lexical, semantic, and visual). The diversification of activation supports the duration of the memory and therefore access to explicit memory [27–29]. This potential projection represents a premise for future research where it will be studied how to improve the memory for verbalized instructions formulated by simple sentences and when it is needed to restore sequential abilities in everyday life, such as brushing teeth, fastening the pants waist, and drying hands [70].

## Figures and Tables

**Table 1:** Demographic data.

	Groups			
	18 Mild AD		18 healthy older adults	
	Means	SD	Means	SD
Years of education	7.1	3.10	7.0	2.80
Age	83.94	2.62	80.12	1.46
MMSE	20.30	4.10	27.30	2.10
Men	22%		17%	
Women	78%		83%	

MMSE: Mini Mental State Examination.

**Table 2:** Recall rates of the second element of the pair with and without sentence context.

	Groups									
	18 mild AD				18 healthy older adults					
Variables	Without sentence		With sentence		Without sentence		With sentence		Means	
	Means	SD	Means	SD	Means	SD	Means	SD	Means	SD
Word/word	5.50	2.56	8.11^∗^	2.85	9.30	2.39	12.66^∗^	1.63	8.89	2.35
Word/picture	8.94^∗^	2.61	11.00^∗^	3.19	11.72^∗^	2.49	14.44^∗^	1.09	11.52	2.34
Means	7.22	2.58	9.55	3.02	10.51	2.44	13.55	1.36		

ANOVA 2 × 2 × 2 was performed; variables encoding type word-word, word-picture, and the presence or absence of sentence context. ^∗^The significance level was set at *p* < .05. A significant main effect in word-picture compared with word-word, and in sentences compared to the null sentence context condition, in both groups was found.
